# Validation of xMAP SARS-CoV-2 Multi-Antigen IgG assay in Nigeria

**DOI:** 10.1371/journal.pone.0266184

**Published:** 2022-04-01

**Authors:** Nnaemeka C. Iriemenam, Fehintola A. Ige, Stacie M. Greby, Augustine Mpamugo, Ado G. Abubakar, Ayuba B. Dawurung, Mudiaga K. Esiekpe, Andrew N. Thomas, Mary U. Okoli, Samuel S. Awala, Blessing N. Ugboaja, Chimaoge C. Achugbu, Ifeanyichukwu Odoh, Felicia D. Nwatu, Temitope Olaleye, Loveth Akayi, Oluwaseun O. Akinmulero, Joseph Dattijo, Edewede Onokevbagbe, Olumide Okunoye, Nwando Mba, Ndidi P. Agala, Mabel Uwandu, Maureen Aniedobe, Kristen A. Stafford, Alash’le Abimiku, Yohhei Hamada, Mahesh Swaminathan, McPaul I. Okoye, Laura C. Steinhardt, Rosemary Audu

**Affiliations:** 1 Division of Global HIV and TB, Center for Global Health, Centers for Disease Control and Prevention, Abuja, Nigeria; 2 Microbiology Department, Center for Human Virology and Genomics, Nigerian Institute of Medical Research, Yaba, Lagos, Nigeria; 3 University of Maryland, Baltimore, Abuja, Nigeria; 4 International Research Center of Excellence, Institute of Human Virology, Abuja, Nigeria; 5 National Reference Laboratory, Nigeria Centre for Disease Control Abuja, Abuja, Nigeria; 6 University of Maryland School of Medicine, Baltimore, MD, United States of America; 7 Institute for Global Health, University College London, London, United Kingdom; 8 Malaria Branch, Division of Parasitic Diseases and Malaria, Center for Global Health, Centers for Disease Control and Prevention, Atlanta, Georgia, United States of America; California State University Fresno, UNITED STATES

## Abstract

**Objective:**

There is a need for reliable serological assays to determine accurate estimates of severe acute respiratory syndrome coronavirus 2 (SARS-CoV-2) seroprevalence. Most single target antigen assays have shown some limitations in Africa. To assess the performance of a multi-antigen assay, we evaluated a commercially available SARS-CoV-2 Multi-Antigen IgG assay for human coronavirus disease 2019 (COVID-19) in Nigeria.

**Methods:**

Validation of the xMAP SARS-CoV-2 Multi-Antigen IgG assay was carried out using well-characterized SARS-CoV-2 reverse transcription polymerase chain reactive positive (97) and pre-COVID-19 pandemic (86) plasma panels. Cross-reactivity was assessed using pre-COVID-19 pandemic plasma specimens (213) from the 2018 Nigeria HIV/AIDS Indicator and Impact Survey (NAIIS).

**Results:**

The overall sensitivity of the xMAP SARS-CoV-2 Multi-Antigen IgG assay was 75.3% [95% CI: 65.8%– 82.8%] and specificity was 99.0% [95% CI: 96.8%– 99.7%]. The sensitivity estimate increased to 83.3% [95% CI: 70.4%– 91.3%] for specimens >14 days post-confirmation of diagnosis. However, using the NAIIS pre-pandemic specimens, the false positivity rate was 1.4% (3/213).

**Conclusions:**

Our results showed overall lower sensitivity and a comparable specificity with the manufacturer’s validation. There appears to be less cross-reactivity with NAIIS pre-pandemic COVID-19 specimens using the xMAP SARS-CoV-2 Multi-Antigen IgG assay. In-country SARS-CoV-2 serology assay validation can help guide the best choice of assays in Africa.

## Introduction

Coronavirus disease 2019 (COVID-19) was first reported in Wuhan, China, in 2019 [1]. The causative agent, severe acute respiratory syndrome coronavirus 2 (SARS-CoV-2), has spread globally, resulting in over three hundred million confirmed cases and over five million deaths as of January 17, 2022 [2]. World Health Organization (WHO) recommends using SARS-CoV-2 serological assays for surveillance in the ongoing pandemic investigation to help understand transmission patterns in various settings [3]. There are many commercially available SARS-CoV-2 immunoassays, some with emergency use authorization (EUA) status [4] and WHO emergency use listing (EUL) status [5] for use in COVID-19 seroprevalence studies.

However, studies conducted in several African countries using in-country specimens showed limited specificity with commercial SARS-CoV-2 assays [6, 7]. With the reported evidence of cross-reactivity from multiple studies in patients co-infected with malaria, endemic in sub-Saharan Africa [6, 8], and other pathogens like HIV [9], SARS-CoV-2 serological tests with more than one target may be preferred, especially when utilized in seroprevalence studies. A previous study showed that serological assays based on one target antigen might not be optimal in low seroprevalence settings [10], and multi-antigen assays using the principle of the multiplex assay may be a more robust, accurate, and reliable serological classification of individuals with prior SARS-CoV-2 infection [11]. Additionally, a cross-sectional serological survey in the Democratic Republic of the Congo that looked at five different SARS-CoV-2 serology tests: two in-house Luminex IgG based assays using recombinant nucleocapsid and spike protein 1, and three commercial assays revealed that a combination of serological tests targeting two or more independent antigens is better to understand the overall serology profile [12].

In-country validation of SARS-CoV-2 assays prior to use is crucial to avoid biased estimates of COVID-19 seroprevalence. WHO recommends that antibody tests for SARS-CoV-2 infection should have a desired sensitivity and specificity of at least 98% and 99%, respectively [13]. In Nigeria, an in-country validation of four SARS-CoV-2 serological assays showed lower sensitivity than manufacturers’ results [14]. Additional testing showed moderate to substantial cross-reactivity levels for two SARS-CoV-2 serological assays, Abbott Architect IgG and Euroimmun NCP [15]. Both of them targeted a single antigen, the nucleocapsid protein. The objective of this study was to validate a commercially available SARS-CoV-2 Multi-Antigen IgG assay for use in serosurveillance studies in Nigeria.

## Materials and methods

### Specimen collection

The description of the sample selection and panel composition used in the validation has been published elsewhere [14, 15]. Briefly, nasal and oropharyngeal swabs (n = 100), as well as the corresponding whole blood specimens from ambulatory participants over 18 years of age who visited the Nigerian Institute of Medical Research (NIMR) drive-through testing center were collected. The time period of sample collection was six months (April to September 2020). Diagnosis of SARS-CoV-2 infection was performed using Cobas® 6800 system (Roche Diagnostics, Basel, Switzerland), and BGI Group (BGI) real-time fluorescent reverse transcription polymerase chain reaction (RT-PCR) methodology on the swab specimens. The RT-PCR procedure was done according to the manufacturer’s instructions.

Whole blood specimens were collected from consented individuals at different time points (0–3, 4–7, 8–14, 15–28, and ≥29 days) after the initial RT-PCR positive result. The whole blood specimens (5–6 ml) were collected in ethylenediaminetetraacetic acid (EDTA) tubes and separated within three hours of blood collection. Centrifugation was done at 4000 rpm for five minutes, and plasma specimens were retrieved into plain collection tubes and frozen at -20°C. The pre-COVID-19 pandemic samples (n = 86) were archived plasma HIV and hepatitis B surface antigen (HBsAg) positive specimens stored at NIMR before October 2019. Also tested were pre-COVID-19 pandemic specimens (n = 213) from the 2018 Nigeria HIV/AIDS Indicator and Impact Survey (NAIIS), stored at the Biorepository of the National Reference Laboratory (NRL), a specimen set that included information on co-infection with malaria. All the 2018 NAIIS stored specimens used in this study were from study participants that provided written consent for their specimens to be used in future testing. Only NAIIS specimens with results for HIV status, malaria infection, and other diseases were included. Previous studies have reported evidence of cross-reactivity in patients co-infected with malaria and other pathogens [6, 8, 9, 15]. The NAIIS specimens, characterized using the multiplex assay, were specifically included for cross-reactivity analysis. All specimens were tested using the xMAP SARS-CoV-2 Multi-Antigen IgG assay at the NRL.

### Laboratory testing

The xMAP SARS-CoV-2 Multi-Antigen IgG assay is a commercially available SARS-CoV-2 IgG assay developed by Luminex. It is a multiplexed microsphere-based assay that measures the presence of IgG antibodies directed against the nucleocapsid protein (NCP), the receptor-binding domain (RBD) of the spike protein, and the S1 subunit of the spike (S) protein of SARS-CoV-2 in human serum or plasma. The assay was done at the NRL according to the manufacturer’s instruction [16]. Briefly, positive and negative kit controls were diluted using wash buffer to a final dilution of 1:20. The plasma specimens were diluted to 1:400 dilutions by performing two 1:20 dilutions. Fifty (50) μl of the diluted specimens and controls were added into the assay plates, and coupled beads were vortexed and transferred into each well. The plates were sealed and incubated in the dark on a shaker at 800 rpm for 60 minutes at room temperature. Then, the plates were washed three times using the wash buffer, and 50 μl of the detection antibody was added to each well, and the plates were incubated for another 60 minutes. The plates were washed two times, and 100 μl of wash buffer was added to each well, mixed, and the plates were read using the Luminex xMAP Magpix System (Luminex Corporation, Austin, USA). Both positive and negative kit controls were added to each assay plate. The controls were monitored for accuracy and assay variation using the kit controls. The positive and negative results were determined using the xMAP Multi IgG CoV-2 Multi-Antigen IgG assay software. The software used an algorithm based on the microsphere counts and median fluorescence intensity (MFI) of controls checked against pre-defined threshold values.

### Ethics approval

Ethical approval was received from the NIMR Institutional Review Board (IRB) and the National Health Research Ethics Committee of Nigeria (NHREC). This activity was reviewed by CDC and was conducted consistent with applicable federal law and CDC policy (45 C.F.R. part 46, 21 C.F.R. part 56; 42 U.S.C. §241(d); 5 U.S.C. §552a; 44 U.S.C. §3501 et seq.).

### Statistical analysis

A specimen was called SARS-CoV-2 IgG positive if the MFI value for the NCP target antigen control was above the set threshold and at least one of the MFI values of the spike protein target antigen controls (S1 or RBD) was above the threshold [16]. Sensitivity and specificity were calculated, with their corresponding 95% confidence intervals (CI), using the SARS-CoV-2 RT-PCR positive and negative panels from NIMR. The confidence intervals were calculated using the Wilson-score method [17]. Additionally, we estimated the sensitivity of the xMAP SARS-CoV-2 Multi-Antigen IgG assay stratified by days of post RT-PCR confirmation of SARS-CoV-2 infection: 0–3 days, 4–7 days, 8–14 days, 15–28 days, and ≥29 days. We also restricted the test sensitivity to samples collected between day 14 and above. Logarithmic transformed MFI values were compared among antibody responses to SARS-CoV-2 IgG negatives and positives using Wilcoxon rank-sum test. Using the multivariable logistic regression model, we estimated the association of false negative with age, sex, and days of post RT-PCR confirmation. Data analysis was performed by IBM SPSS Statistics version 21.0 (IBM Corporation, NY, USA), GraphPad Prism (GraphPad Software, San Diego, USA) and Microsoft Excel (Microsoft Corporations, Redmond, USA).

## Results

In total, 100 SARS-CoV-2 RT-PCR positive specimens and 299 pre-COVID-19 pandemic specimens from NIMR and NAIIS were used in the evaluation of sensitivity and specificity. However, 3 SARS-CoV-2 RT-PCR positive specimens were excluded from the analysis due to no specimen (1 specimen) and “no call” status (did not meet the target limit) after repeat testing (2 specimens). The characteristics of SARS-CoV-2 RT-PCR positive samples are shown in Table 1.

**Table 1:** Characteristics of SARS-CoV-2 RT-PCR positive samples.

Characteristics	N	%
**Age (Years)**		
** Median (IQR)**	36.0 [29.3–42.0]	
**Sex**		
** Male**	43	44.3
** Female**	47	48.5
** Missing**	7	7.2
**Days post RT-PCR confirmation of SARS-CoV-2 infection**		
** 0–3**	10	10.3
** 4–7**	12	12.4
** 8–14**	27	27.8
** 15–28**	21	21.6
** ≥29**	27	27.8

N = number, % = percentage, IQR = interquartile range.

The median age was 36 years (interquartile range [IQR], 29.3 and 42.0 years), and 44.3% were males, 48.5% were females, and 7.2% had missing information on sex. Most samples were taken after 8–14 (27.8%) and ≥29 days (27.8%) post RT-PCR confirmation of SARS-CoV-2 infection. Of the 97 SARS-CoV-2 RT-PCR-positive specimens, 24.7% (24/97) tested negative, and 75.3% (73/97) tested positive with the xMAP SARS-CoV-2 Multi-Antigen IgG assay algorithm.

The 73 positive specimens were positive for both the NCP and RBD targets; 3 of these specimens were also positive for the third target (S1). All the 86 (100%) pre-COVID-19 NIMR pandemic specimens also tested negative with the xMAP SARS-CoV-2 Multi-Antigen IgG assay. The percentage coefficient of variation for reproducibility ranged from 21.1%-31.5% for the negative control and 22.1%-28% for the positive control.

### Sensitivity and specificity

The overall sensitivity of the xMAP SARS-CoV-2 Multi-Antigen IgG assay using Nigeria specimens was 75.3% [95% CI: 65.8%– 82.8%], and specificity was 99.0% [95% CI: 96.8%– 99.7%] (Table 2). The sensitivity increased from samples taken between 4–7 days post RT-PCR confirmation, then decreased between 15 to 28 days, and increased afterwards (Table 2).

**Table 2:** Sensitivity by days post RT-PCR confirmation and specificity of xMAP SARS-CoV-2 Multi-Antigen IgG Assay using the NIMR panel and 2018 NAIIS pre-pandemic samples, Nigeria, 2021.

Days post RT-PCR Confirmation	True Positive (N)	False Negative (N)	Sub-Total (N)	Sensitivity, %	95% CI	True Negative (N)	False Positive (N)	Sub-Total (N)	Specificity, %	95% CI	Total
**Overall**	73	24	97	75.3	65.8–82.8						
**0–3**	3	7	10	30.0	8.1–64.6	296	3	299	99.0	96.8–99.7	
**4–7**	8	4	12	66.7	35.4–88.7						
**8–14**	22	5	27	81.5	61.3–93.0						
**15–28**	16	5	21	76.2	52.5–90.9						
**>29**	24	3	27	88.9	69.7–97.1						
**Total**			**97**					**299**			**396**

CI = confidence interval, N = number.

When stratified by days of post confirmation of SARS-CoV-2 diagnosis by RT-PCR, the sensitivity estimate increased from 67.3% [95% CI: 53.4%– 78.8%] for specimens ≤14 days post RT-PCR confirmation to 83.3% [95% CI: 70.4%– 91.3%] for specimens taken more than 14 days after RT-PCR confirmation. The overall specificity using the two groups of pre-COVID-19 pandemic specimens (86 from NIMR and 213 from NAIIS) was 99.0% [95% CI: 96.8%– 99.7%] (Table 2). Using 0–3 days as the indicator in the multivariable logistic regression model and adjusting for age, sex, and days of post RT-PCR confirmation of SARS-CoV-2 infection, there were no association with false negative results.

### Cross-reactive SARS-CoV-2 antibodies to the 2018 NAIIS pre-pandemic specimens

**Fig 1:**
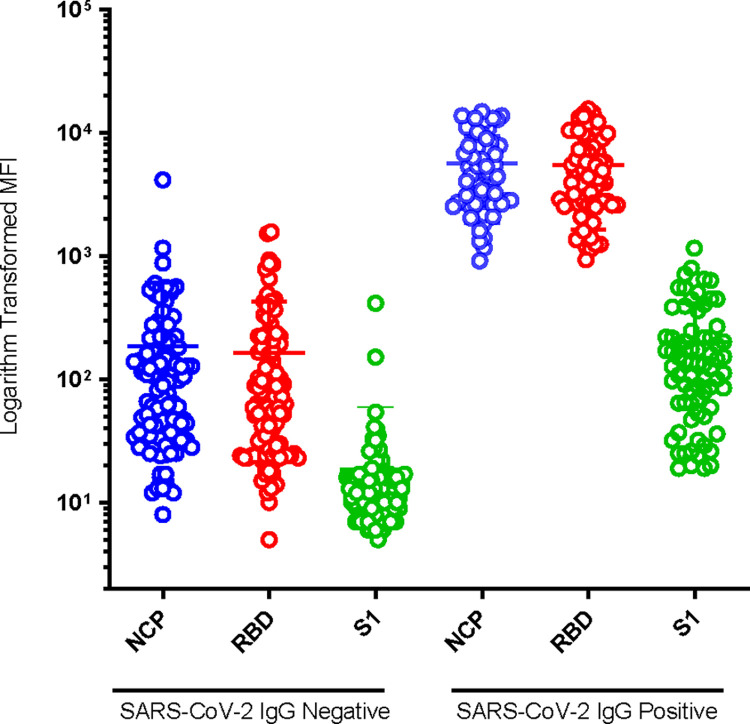
Dot plot indicating logarithmic transformed MFI levels of antibody responses to SARS-CoV-2 IgG negative and positive specimens of the 2018 NAIIS pre-pandemic samples, Nigeria, 2021. NCP = nucleocapsid, RBD = receptor-binding domain, S1 = spike.

Fig 1 shows the graphical presentation of MFI levels of antibody responses to SARS-CoV-2 IgG negative and positive specimens of the 2018 NAIIS pre-COVID-19 pandemic samples. The MFI of the SARS-CoV-2 positive targets (NCP, RBD, and S1) were significantly higher (p<0.001) than that of the SARS-CoV-2 negative (Fig 1).

The highest level of cross-reactive antibodies, based on the microsphere counts and MFI, was observed with NCP 16.4% (35/213), followed by the RBD 4.7% (10/213), and the least with S1 0.5% (1/213). The interpretation of final positivity results based on the Luminex xMAP SARS-CoV-2 Multi-Antigen IgG assay algorithm indicated that 1.4% (3/213) of the 2018 NAIIS pre-COVID-19 pandemic specimens were false positive.

## Discussion

This study found an overall sensitivity of 75.3% for the xMAP SARS-CoV-2 Multi-Antigen IgG assay., However, when restricted to specimens taken more than two weeks post confirmation of infection, the sensitivity increased to 83.3%. The sensitivity values appear lower than the manufacturer’s reported sensitivity of 96.3%. However, our specificity estimate was 99.0% (using all samples in the study), which is comparable to the manufacturer’s reported specificity of 99.3%. When compared to previous validations of SARS-CoV-2 immunoassays in Nigeria using Abbott Architect SARS-COV-2 IgG, Euroimmun Anti-SARS-CoV-2 NCP IgG, Euroimmun Spike SARS-CoV-2 IgG, and Omega Mologic COVID-19 IgG [14], the sensitivity of xMAP SARS-CoV-2 Multi-Antigen IgG assay was found to be higher than that of other assays, which ranged from 73.7% to 76.9% in specimens taken >14 days post RTPCR confirmation of infection. The false positivity rate of 1.4% observed in this study for xMAP SARS-CoV-2 Multi-Antigen IgG assay, appears lower than the rates from a previous cross-reactivity study of two SARS-CoV-2 serological assays (Euroimmun NCP and Abbott Architect IgG) using the 2018 pre-COVID-19 pandemic specimens, which indicated false positivity rates of 17.8% and 6.1%, respectively [15].

The importance of in-country validations of assays for infectious diseases has been previously documented [18]. Since the beginning of the SARS-CoV-2 pandemic, few validations have been published from countries in Africa. Importantly, appropriate validation of SARS-CoV-2 serological assays is required for countries preparing for COVID-19 seroprevalence studies, owing to differences in assay methodology [19], selection of viral antigens and isotypes of antibodies, individual variance, and antibody levels fluctuations [20]. In countries where malaria is endemic, false positive results may be likely due to non-specific immune responses, leading to overestimates and misinterpretation of SARS-CoV-2 infection [6]. Therefore, using a single target antigen assay in these malaria-endemic regions may not be optimal for accurate SARS-CoV-2 seroprevalence estimates. Potential cross-reactivity mechanisms may be multi-factorial, but previous *Plasmodium* infection and exposure to other human CoVs may induce cross-reactive antibodies against SARS-CoV-2 infection [21]. Our results agree with previous studies indicating that combining multiple markers had the highest sensitivity and specificity in detecting low-level antibody responses to SARS-CoV-2 antigens [22, 23] and extend these findings to a malaria-endemic region.

The NCP is the most abundant protein produced within infected cells and is highly antigenic [24]. In our study using the 2018 NAIIS pre-COVID-19 pandemic specimens, the highest level of cross-reactivity was observed with the NCP target followed by the RBD and then S1. In addition, a recent report from a U.S. population showed that the most cross-reactive target was the NCP, followed by S1 and the RBD [25]. Thus, it is possible that some individuals had SARS-CoV-2 cross-reactive antibodies prior to the COVID-19 pandemic and these cross-reactive antibodies might increase the likelihood of false positivity. Combining multiple targets increases the sensitivity of SARS-CoV-2 immunoassays and reduces the chances of false positivity. Another essential feature of multiplex assays is capturing a broader breadth of responses against multiple targets, which is not possible using a single target assay [26].

An additional advantage is the small specimen volume required for the multiplex assay, and multiple analytes analyses could be performed with a small amount of specimen. One limitation to this study is that we do not have information on other coronaviruses possibly circulating in this population. In addition, the positive specimens’ population were ambulatory individuals and not patients with severe or critical COVID-19. A previous study showed that antibody response levels increased with increasing severity of illness, including hospitalization [27].

In conclusion, our study showed that a SARS-CoV-2 multi-antigen multiplex assay had lower cross-reactivity and comparable sensitivity with previous single target antigen assay validations in Nigeria. For countries preparing for COVID-19 serosurveillance, it is vital to perform in-country SARS-CoV-2 serology assay validation as the assay needs to reflect the serologic profile of the population being tested.
