# Correction: Validation of xMAP SARS-CoV-2 Multi-Antigen IgG assay in Nigeria

**DOI:** 10.1371/journal.pone.0333990

**Published:** 2025-10-07

**Authors:** The PLOS One Staff

## Notice of Republication

This article was republished on September 17, 2025, to correct an error in the twelfth author’s name that was introduced during the typesetting process. The correct name is: Chimaoge C. Achugbu. The publisher apologizes for the error. Please download this article again to view the correct version. The originally published, uncorrected article and the republished, corrected articles are provided here for reference.

## Supporting information

S1 File Originally published, uncorrected article. (PDF)

S2 File Republished, corrected article. (PDF)
