# Supplementary material for: Correction: Validation of xMAP SARS-CoV-2 Multi-Antigen IgG assay in Nigeria
Source: PLoS One. 2025 Oct 7;20(10):e0333990. doi: 10.1371/journal.pone.0333990 (PMC12503247; doi:10.1371/journal.pone.0333990)
Supplement: S1 File — (PDF) [file pone.0333990.s001.pdf]

PLOS ONE

RESEARCH ARTICLE

Validation of xMAP SARS-CoV-2 Multi-Antigen IgG assay in Nigeria

Nnaemeka C. Iriemenam[1]*, Fehintola A. Ige[2], Stacie M. Greby[1], Augustine Mpamugo[3], Ado G. Abubakar[4], Ayuba B. Dawurung[4], Mudiaga K. Esiekpe[4], Andrew N. Thomas[4], Mary U. Okoli[5], Samuel S. Awala[4], Blessing N. Ugboaja[4], Chicago C. Achugbu[5], Ifeanyichukwu Odoh[4], Felicia D. Nwatu[4], Temitope Olaleye[4], Loveth Akayi[4], Oluwaseun O. Akinmulero[4], Joseph Dattijo[4], Edewede Onokevbagbe[3], Olumide Okunoye[1], Nwando Mba[5], Ndidi P. Agala[4], Mabel Uwandu[2], Maureen Aniedobe[2], Kristen A. Stafford[6], Alash'le Abimiku[4,6], Yohhei Hamada[7], Mahesh Swaminathan[1], McPaul I. Okoye[1], Laura C. Steinhardt[8], Rosemary Audu[2]

1 Division of Global HIV and TB, Center for Global Health, Centers for Disease Control and Prevention, Abuja, Nigeria, **2** Microbiology Department, Center for Human Virology and Genomics, Nigerian Institute of Medical Research, Yaba, Lagos, Nigeria, **3** University of Maryland, Baltimore, Abuja, Nigeria, **4** International Research Center of Excellence, Institute of Human Virology, Abuja, Nigeria, **5** National Reference Laboratory, Nigeria Centre for Disease Control Abuja, Abuja, Nigeria, **6** University of Maryland School of Medicine, Baltimore, MD, United States of America, **7** Institute for Global Health, University College London, London, United Kingdom, **8** Malaria Branch, Division of Parasitic Diseases and Malaria, Center for Global Health, Centers for Disease Control and Prevention, Atlanta, Georgia, United States of America

* NIriemenam@cdc.gov

Check for updates

OPEN ACCESS

Citation: Iriemenam NC, Ige FA, Greby SM, Mpamugo A, Abubakar AG, Dawurung AB, et al. (2022) Validation of xMAP SARS-CoV-2 Multi-Antigen IgG assay in Nigeria. PLoS ONE 17(4): e0266184. https://doi.org/10.1371/journal.pone.0266184

Editor: Viswanathan V Krishnan, California State University Fresno, UNITED STATES

Received: September 8, 2021

Accepted: March 15, 2022

Published: April 1, 2022

Copyright: This is an open access article, free of all copyright, and may be freely reproduced, distributed, transmitted, modified, built upon, or otherwise used by anyone for any lawful purpose. The work is made available under the Creative Commons CC0 public domain dedication.

Data Availability Statement: All relevant data are within the manuscript.

Funding: NAIIS was supported by the President's Emergency Plan for AIDS Relief (PEPFAR) through CDC under the cooperative agreement #U2GGH002108 to UMB and by the Global Funds to Fight AIDS, Tuberculosis, and Malaria through NACA, under the contract # NGA-H-NACA to UMB. The funders had no role in study design, data collection and analysis, decision to publish, or preparation of the manuscript. Disclaimer The

Abstract

Objective

There is a need for reliable serological assays to determine accurate estimates of severe acute respiratory syndrome coronavirus 2 (SARS-CoV-2) seroprevalence. Most single target antigen assays have shown some limitations in Africa. To assess the performance of a multi-antigen assay, we evaluated a commercially available SARS-CoV-2 Multi-Antigen IgG assay for human coronavirus disease 2019 (COVID-19) in Nigeria.

Methods

Validation of the xMAP SARS-CoV-2 Multi-Antigen IgG assay was carried out using well-characterized SARS-CoV-2 reverse transcription polymerase chain reactive positive (97) and pre-COVID-19 pandemic (86) plasma panels. Cross-reactivity was assessed using pre-COVID-19 pandemic plasma specimens (213) from the 2018 Nigeria HIV/AIDS Indicator and Impact Survey (NAIIS).

Results

The overall sensitivity of the xMAP SARS-CoV-2 Multi-Antigen IgG assay was 75.3% [95% CI: 65.8%– 82.8%] and specificity was 99.0% [95% CI: 96.8%– 99.7%]. The sensitivity estimate increased to 83.3% [95% CI: 70.4%– 91.3%] for specimens >14 days post-confirmation of diagnosis. However, using the NAIIS pre-pandemic specimens, the false positivity rate was 1.4% (3/213).

findings and conclusions in this report are those of the authors and do not necessarily represent the views of the Centers for Disease Control and Prevention. The findings and conclusions of this report are those of the authors and do not necessarily represent the official position of the NAIIS Group. Luminex xMAP SARS-CoV-2 Multi-Antigen IgG assay is for informational purposes and does not constitute endorsement by the Centers for Disease Control and Prevention."

Competing interests: The authors have declared that no competing interests exist.

Conclusions

Our results showed overall lower sensitivity and a comparable specificity with the manufacturer's validation. There appears to be less cross-reactivity with NAIIS pre-pandemic COVID-19 specimens using the xMAP SARS-CoV-2 Multi-Antigen IgG assay. In-country SARS-CoV-2 serology assay validation can help guide the best choice of assays in Africa.

Introduction

Coronavirus disease 2019 (COVID-19) was first reported in Wuhan, China, in 2019 [1]. The causative agent, severe acute respiratory syndrome coronavirus 2 (SARS-CoV-2), has spread globally, resulting in over three hundred million confirmed cases and over five million deaths as of January 17, 2022 [2]. World Health Organization (WHO) recommends using SARS-CoV-2 serological assays for surveillance in the ongoing pandemic investigation to help understand transmission patterns in various settings [3]. There are many commercially available SARS-CoV-2 immunoassays, some with emergency use authorization (EUA) status [4] and WHO emergency use listing (EUL) status [5] for use in COVID-19 seroprevalence studies.

However, studies conducted in several African countries using in-country specimens showed limited specificity with commercial SARS-CoV-2 assays [6, 7]. With the reported evidence of cross-reactivity from multiple studies in patients co-infected with malaria, endemic in sub-Saharan Africa [6, 8], and other pathogens like HIV [9], SARS-CoV-2 serological tests with more than one target may be preferred, especially when utilized in seroprevalence studies. A previous study showed that serological assays based on one target antigen might not be optimal in low seroprevalence settings [10], and multi-antigen assays using the principle of the multiplex assay may be a more robust, accurate, and reliable serological classification of individuals with prior SARS-CoV-2 infection [11]. Additionally, a cross-sectional serological survey in the Democratic Republic of the Congo that looked at five different SARS-CoV-2 serology tests: two in-house Luminex IgG based assays using recombinant nucleocapsid and spike protein 1, and three commercial assays revealed that a combination of serological tests targeting two or more independent antigens is better to understand the overall serology profile [12].

In-country validation of SARS-CoV-2 assays prior to use is crucial to avoid biased estimates of COVID-19 seroprevalence. WHO recommends that antibody tests for SARS-CoV-2 infection should have a desired sensitivity and specificity of at least 98% and 99%, respectively [13]. In Nigeria, an in-country validation of four SARS-CoV-2 serological assays showed lower sensitivity than manufacturers' results [14]. Additional testing showed moderate to substantial cross-reactivity levels for two SARS-CoV-2 serological assays, Abbott Architect IgG and Euroimmun NCP [15]. Both of them targeted a single antigen, the nucleocapsid protein. The objective of this study was to validate a commercially available SARS-CoV-2 Multi-Antigen IgG assay for use in serosurveillance studies in Nigeria.

Materials and methods

Specimen collection

The description of the sample selection and panel composition used in the validation has been published elsewhere [14, 15]. Briefly, nasal and oropharyngeal swabs (n = 100), as well as the corresponding whole blood specimens from ambulatory participants over 18 years of age who

visited the Nigerian Institute of Medical Research (NIMR) drive-through testing center were collected. The time period of sample collection was six months (April to September 2020). Diagnosis of SARS-CoV-2 infection was performed using Cobas® 6800 system (Roche Diagnostics, Basel, Switzerland), and BGI Group (BGI) real-time fluorescent reverse transcription polymerase chain reaction (RT-PCR) methodology on the swab specimens. The RT-PCR procedure was done according to the manufacturer's instructions.

Whole blood specimens were collected from consented individuals at different time points (0–3, 4–7, 8–14, 15–28, and ≥29 days) after the initial RT-PCR positive result. The whole blood specimens (5–6 ml) were collected in ethylenediaminetetraacetic acid (EDTA) tubes and separated within three hours of blood collection. Centrifugation was done at 4000 rpm for five minutes, and plasma specimens were retrieved into plain collection tubes and frozen at -20˚C. The pre-COVID-19 pandemic samples (n = 86) were archived plasma HIV and hepatitis B surface antigen (HBsAg) positive specimens stored at NIMR before October 2019. Also tested were pre-COVID-19 pandemic specimens (n = 213) from the 2018 Nigeria HIV/AIDS Indicator and Impact Survey (NAIIS), stored at the Biorepository of the National Reference Laboratory (NRL), a specimen set that included information on co-infection with malaria. All the 2018 NAIIS stored specimens used in this study were from study participants that provided written consent for their specimens to be used in future testing. Only NAIIS specimens with results for HIV status, malaria infection, and other diseases were included. Previous studies have reported evidence of cross-reactivity in patients co-infected with malaria and other pathogens [6, 8, 9, 15]. The NAIIS specimens, characterized using the multiplex assay, were specifically included for cross-reactivity analysis. All specimens were tested using the xMAP SARS-CoV-2 Multi-Antigen IgG assay at the NRL.

Laboratory testing

The xMAP SARS-CoV-2 Multi-Antigen IgG assay is a commercially available SARS-CoV-2 IgG assay developed by Luminex. It is a multiplexed microsphere-based assay that measures the presence of IgG antibodies directed against the nucleocapsid protein (NCP), the receptor-binding domain (RBD) of the spike protein, and the S1 subunit of the spike (S) protein of SARS-CoV-2 in human serum or plasma. The assay was done at the NRL according to the manufacturer's instruction [16]. Briefly, positive and negative kit controls were diluted using wash buffer to a final dilution of 1:20. The plasma specimens were diluted to 1:400 dilutions by performing two 1:20 dilutions. Fifty (50) μl of the diluted specimens and controls were added into the assay plates, and coupled beads were vortexed and transferred into each well. The plates were sealed and incubated in the dark on a shaker at 800 rpm for 60 minutes at room temperature. Then, the plates were washed three times using the wash buffer, and 50 μl of the detection antibody was added to each well, and the plates were incubated for another 60 minutes. The plates were washed two times, and 100 μl of wash buffer was added to each well, mixed, and the plates were read using the Luminex xMAP Magpix System (Luminex Corporation, Austin, USA). Both positive and negative kit controls were added to each assay plate. The controls were monitored for accuracy and assay variation using the kit controls. The positive and negative results were determined using the xMAP Multi IgG CoV-2 Multi-Antigen IgG assay software. The software used an algorithm based on the microsphere counts and median fluorescence intensity (MFI) of controls checked against pre-defined threshold values.

Ethics approval

Ethical approval was received from the NIMR Institutional Review Board (IRB) and the National Health Research Ethics Committee of Nigeria (NHREC). This activity was reviewed

by CDC and was conducted consistent with applicable federal law and CDC policy (45 C.F.R. part 46, 21 C.F.R. part 56; 42 U.S.C. §241(d); 5 U.S.C. §552a; 44 U.S.C. §3501 et seq.).

Statistical analysis

A specimen was called SARS-CoV-2 IgG positive if the MFI value for the NCP target antigen control was above the set threshold and at least one of the MFI values of the spike protein target antigen controls (S1 or RBD) was above the threshold [16]. Sensitivity and specificity were calculated, with their corresponding 95% confidence intervals (CI), using the SARS-CoV-2 RT-PCR positive and negative panels from NIMR. The confidence intervals were calculated using the Wilson-score method [17]. Additionally, we estimated the sensitivity of the xMAP SARS-CoV-2 Multi-Antigen IgG assay stratified by days of post RT-PCR confirmation of SARS-CoV-2 infection: 0–3 days, 4–7 days, 8–14 days, 15–28 days, and ≥29 days. We also restricted the test sensitivity to samples collected between day 14 and above. Logarithmic transformed MFI values were compared among antibody responses to SARS-CoV-2 IgG negatives and positives using Wilcoxon rank-sum test. Using the multivariable logistic regression model, we estimated the association of false negative with age, sex, and days of post RT-PCR confirmation. Data analysis was performed by IBM SPSS Statistics version 21.0 (IBM Corporation, NY, USA), GraphPad Prism (GraphPad Software, San Diego, USA) and Microsoft Excel (Microsoft Corporations, Redmond, USA).

Results

In total, 100 SARS-CoV-2 RT-PCR positive specimens and 299 pre-COVID-19 pandemic specimens from NIMR and NAIIS were used in the evaluation of sensitivity and specificity. However, 3 SARS-CoV-2 RT-PCR positive specimens were excluded from the analysis due to no specimen (1 specimen) and "no call" status (did not meet the target limit) after repeat testing (2 specimens). The characteristics of SARS-CoV-2 RT-PCR positive samples are shown in Table 1.

The median age was 36 years (interquartile range [IQR], 29.3 and 42.0 years), and 44.3% were males, 48.5% were females, and 7.2% had missing information on sex. Most samples were taken after 8–14 (27.8%) and ≥29 days (27.8%) post RT-PCR confirmation of SARS-CoV-2 infection. Of the 97 SARS-CoV-2 RT-PCR-positive specimens, 24.7% (24/97) tested negative,

Table 1. Characteristics of SARS-CoV-2 RT-PCR positive samples.

Characteristics	N	%
**Age (Years)**		
**Median (IQR)**	36.0 [29.3–42.0]	
**Sex**		
**Male**	43	44.3
**Female**	47	48.5
**Missing**	7	7.2
**Days post RT-PCR confirmation of SARS-CoV-2 infection**		
**0–3**	10	10.3
**4–7**	12	12.4
**8–14**	27	27.8
**15–28**	21	21.6
**≥29**	27	27.8

N = number, % = percentage, IQR = interquartile range.

https://doi.org/10.1371/journal.pone.0266184.t001

and 75.3% (73/97) tested positive with the xMAP SARS-CoV-2 Multi-Antigen IgG assay algorithm.

The 73 positive specimens were positive for both the NCP and RBD targets; 3 of these specimens were also positive for the third target (S1). All the 86 (100%) pre-COVID-19 NIMR pandemic specimens also tested negative with the xMAP SARS-CoV-2 Multi-Antigen IgG assay. The percentage coefficient of variation for reproducibility ranged from 21.1%-31.5% for the negative control and 22.1%-28% for the positive control.

Sensitivity and specificity

The overall sensitivity of the xMAP SARS-CoV-2 Multi-Antigen IgG assay using Nigeria specimens was 75.3% [95% CI: 65.8%– 82.8%], and specificity was 99.0% [95% CI: 96.8%– 99.7%] (Table 2). The sensitivity increased from samples taken between 4–7 days post RT-PCR confirmation, then decreased between 15 to 28 days, and increased afterwards (Table 2).

When stratified by days of post confirmation of SARS-CoV-2 diagnosis by RT-PCR, the sensitivity estimate increased from 67.3% [95% CI: 53.4%– 78.8%] for specimens $\leq$14 days post RT-PCR confirmation to 83.3% [95% CI: 70.4%– 91.3%] for specimens taken more than 14 days after RT-PCR confirmation. The overall specificity using the two groups of pre-COVID-19 pandemic specimens (86 from NIMR and 213 from NAIIS) was 99.0% [95% CI: 96.8%– 99.7%] (Table 2). Using 0–3 days as the indicator in the multivariable logistic regression model and adjusting for age, sex, and days of post RT-PCR confirmation of SARS-CoV-2 infection, there were no association with false negative results.

Cross-reactive SARS-CoV-2 antibodies to the 2018 NAIIS pre-pandemic specimens

Fig 1 shows the graphical presentation of MFI levels of antibody responses to SARS-CoV-2 IgG negative and positive specimens of the 2018 NAIIS pre-COVID-19 pandemic samples. The MFI of the SARS-CoV-2 positive targets (NCP, RBD, and S1) were significantly higher ($p<0.001$) than that of the SARS-CoV-2 negative (Fig 1).

The highest level of cross-reactive antibodies, based on the microsphere counts and MFI, was observed with NCP 16.4% (35/213), followed by the RBD 4.7% (10/213), and the least with

Table 2. Sensitivity by days post RT-PCR confirmation and specificity of xMAP SARS-CoV-2 Multi-Antigen IgG Assay using the NIMR panel and 2018 NAIIS pre-pandemic samples, Nigeria, 2021.

Days post RT-PCR Confirmation	True Positive (N)	False Negative (N)	Sub-Total (N)	Sensitivity, %	95% CI	True Negative (N)	False Positive (N)	Sub-Total (N)	Specificity, %	95% CI	Total
**Overall**	73	24	97	75.3	65.8–82.8						
**0–3**	3	7	10	30.0	8.1–64.6	296	3	299	99.0	96.8–99.7	
**4–7**	8	4	12	66.7	35.4–88.7						
**8–14**	22	5	27	81.5	61.3–93.0						
**15–28**	16	5	21	76.2	52.5–90.9						
**>29**	24	3	27	88.9	69.7–97.1						
**Total**			**97**					**299**			**396**

CI = confidence interval, N = number.

https://doi.org/10.1371/journal.pone.0266184.t002

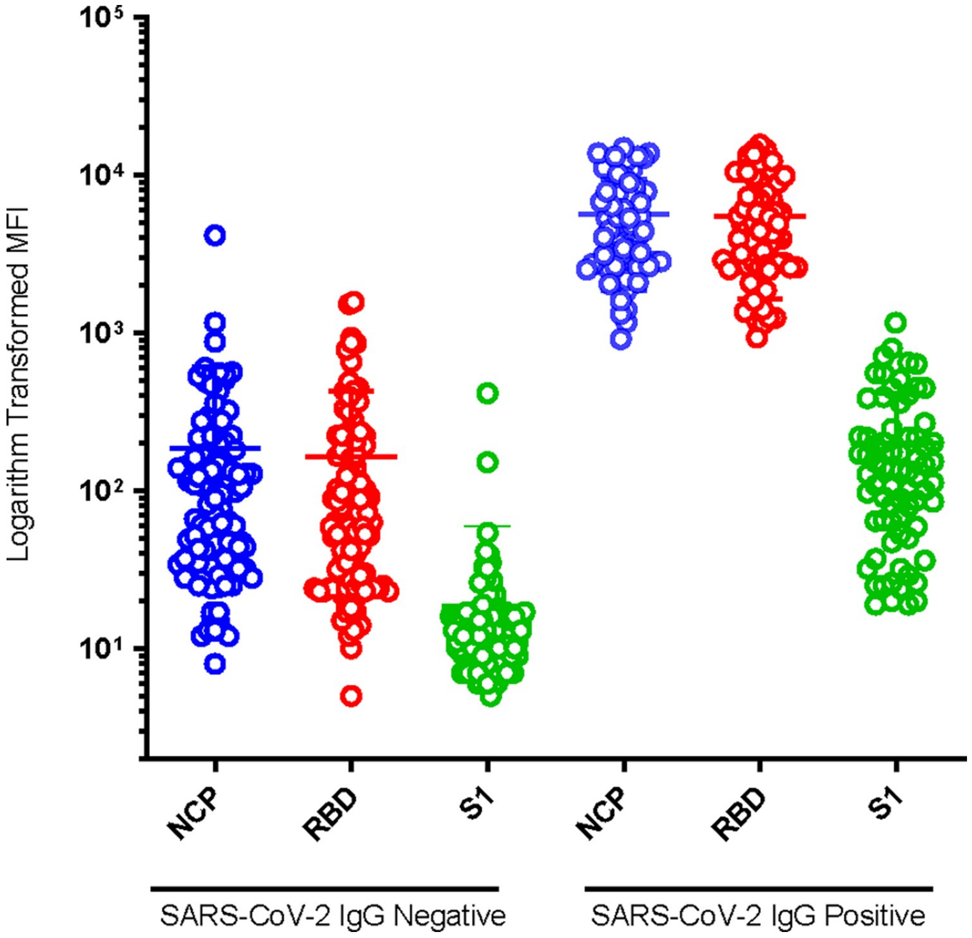

Fig 1. Dot plot indicating logarithmic transformed MFI levels of antibody responses to SARS-CoV-2 IgG negative and positive specimens of the 2018 NAIIS pre-pandemic samples, Nigeria, 2021. NCP = nucleocapsid, RBD = receptor-binding domain, S1 = spike.

https://doi.org/10.1371/journal.pone.0266184.g001

S1 0.5% (1/213). The interpretation of final positivity results based on the Luminex xMAP SARS-CoV-2 Multi-Antigen IgG assay algorithm indicated that 1.4% (3/213) of the 2018 NAIIS pre-COVID-19 pandemic specimens were false positive.

Discussion

This study found an overall sensitivity of 75.3% for the xMAP SARS-CoV-2 Multi-Antigen IgG assay., However, when restricted to specimens taken more than two weeks post confirmation of infection, the sensitivity increased to 83.3%. The sensitivity values appear lower than the manufacturer's reported sensitivity of 96.3%. However, our specificity estimate was 99.0% (using all samples in the study), which is comparable to the manufacturer's reported specificity of 99.3%. When compared to previous validations of SARS-CoV-2 immunoassays in Nigeria using Abbott Architect SARS-COV-2 IgG, Euroimmun Anti-SARS-CoV-2 NCP IgG, Euroimmun Spike SARS-CoV-2 IgG, and Omega Mologic COVID-19 IgG [14], the sensitivity of xMAP SARS-CoV-2 Multi-Antigen IgG assay was found to be higher than that of other assays, which ranged from 73.7% to 76.9% in specimens taken >14 days post RTPCR confirmation of infection. The false positivity rate of 1.4% observed in this study for xMAP SARS-CoV-2 Multi-Antigen IgG assay, appears lower than the rates from a previous cross-reactivity study of

two SARS-CoV-2 serological assays (Euroimmun NCP and Abbott Architect IgG) using the 2018 pre-COVID-19 pandemic specimens, which indicated false positivity rates of 17.8% and 6.1%, respectively [15].

The importance of in-country validations of assays for infectious diseases has been previously documented [18]. Since the beginning of the SARS-CoV-2 pandemic, few validations have been published from countries in Africa. Importantly, appropriate validation of SARS-CoV-2 serological assays is required for countries preparing for COVID-19 seroprevalence studies, owing to differences in assay methodology [19], selection of viral antigens and isotypes of antibodies, individual variance, and antibody levels fluctuations [20]. In countries where malaria is endemic, false positive results may be likely due to non-specific immune responses, leading to overestimates and misinterpretation of SARS-CoV-2 infection [6]. Therefore, using a single target antigen assay in these malaria-endemic regions may not be optimal for accurate SARS-CoV-2 seroprevalence estimates. Potential cross-reactivity mechanisms may be multi-factorial, but previous *Plasmodium* infection and exposure to other human CoVs may induce cross-reactive antibodies against SARS-CoV-2 infection [21]. Our results agree with previous studies indicating that combining multiple markers had the highest sensitivity and specificity in detecting low-level antibody responses to SARS-CoV-2 antigens [22, 23] and extend these findings to a malaria-endemic region.

The NCP is the most abundant protein produced within infected cells and is highly antigenic [24]. In our study using the 2018 NAIIS pre-COVID-19 pandemic specimens, the highest level of cross-reactivity was observed with the NCP target followed by the RBD and then S1. In addition, a recent report from a U.S. population showed that the most cross-reactive target was the NCP, followed by S1 and the RBD [25]. Thus, it is possible that some individuals had SARS-CoV-2 cross-reactive antibodies prior to the COVID-19 pandemic and these cross-reactive antibodies might increase the likelihood of false positivity. Combining multiple targets increases the sensitivity of SARS-CoV-2 immunoassays and reduces the chances of false positivity. Another essential feature of multiplex assays is capturing a broader breadth of responses against multiple targets, which is not possible using a single target assay [26].

An additional advantage is the small specimen volume required for the multiplex assay, and multiple analytes analyses could be performed with a small amount of specimen. One limitation to this study is that we do not have information on other coronaviruses possibly circulating in this population. In addition, the positive specimens' population were ambulatory individuals and not patients with severe or critical COVID-19. A previous study showed that antibody response levels increased with increasing severity of illness, including hospitalization [27].

In conclusion, our study showed that a SARS-CoV-2 multi-antigen multiplex assay had lower cross-reactivity and comparable sensitivity with previous single target antigen assay validations in Nigeria. For countries preparing for COVID-19 serosurveillance, it is vital to perform in-country SARS-CoV-2 serology assay validation as the assay needs to reflect the serologic profile of the population being tested.

Acknowledgments

The authors acknowledge the contribution of valuable specimens by the NAIIS Group, including the Federal Ministry of Health (FMoH), National Agency for the Control of AIDS (NACA), National Population Commission (NPopC), National Bureau of Statistics (NBS), U. S. Centers for Disease Control and Prevention (CDC), The Global Funds to Fight AIDS, Tuberculosis, and Malaria, University of Maryland Baltimore (UMB), ICF International, African Field Epidemiology Network (AFENET), University of Washington (UW), the Joint

United Nations Programme on HIV and AIDS (UNAIDS), World Health Organization (WHO), and United Nations Children's Fund (UNICEF). The authors thank Dr. Molebogeng X. Rangaka of the University College London for her immense technical assistance. The authors also thank the people of Nigeria who provided specimens.

Author Contributions

Conceptualization: Nnaemeka C. Iriemenam, Fehintola A. Ige, Stacie M. Greby, Mahesh Swaminathan, McPaul I. Okoye, Laura C. Steinhardt, Rosemary Audu.

Data curation: Nnaemeka C. Iriemenam, Fehintola A. Ige, Temitope Olaleye, Olumide Okunoye, Laura C. Steinhardt, Rosemary Audu.

Formal analysis: Nnaemeka C. Iriemenam, Yohhei Hamada, Laura C. Steinhardt.

Funding acquisition: Nnaemeka C. Iriemenam, Stacie M. Greby, Mahesh Swaminathan, McPaul I. Okoye, Laura C. Steinhardt.

Investigation: Nnaemeka C. Iriemenam, Fehintola A. Ige, Augustine Mpamugo, Ado G. Abubakar, Ayuba B. Dawurung, Mudiaga K. Esiekpe, Andrew N. Thomas, Mary U. Okoli, Samuel S. Awala, Blessing N. Ugboaja, Chicago C. Achugbu, Ifeanyichukwu Odoh, Felicia D. Nwatu, Temitope Olaleye, Loveth Akayi, Oluwaseun O. Akinmulero, Joseph Dattijo, Edewede Onokevbagbe, Nwando Mba, Ndidi P. Agala, Mabel Uwandu, Maureen Aniedobe, Kristen A. Stafford, Alash'le Abimiku, Yohhei Hamada, McPaul I. Okoye, Rosemary Audu.

Methodology: Nnaemeka C. Iriemenam, Fehintola A. Ige, Augustine Mpamugo, Ado G. Abubakar, Ayuba B. Dawurung, Mudiaga K. Esiekpe, Andrew N. Thomas, Mary U. Okoli, Samuel S. Awala, Blessing N. Ugboaja, Chicago C. Achugbu, Ifeanyichukwu Odoh, Felicia D. Nwatu, Temitope Olaleye, Loveth Akayi, Oluwaseun O. Akinmulero, Joseph Dattijo, Edewede Onokevbagbe, Olumide Okunoye, Nwando Mba, Ndidi P. Agala, Mabel Uwandu, Maureen Aniedobe, Alash'le Abimiku, Yohhei Hamada, McPaul I. Okoye, Rosemary Audu.

Project administration: Nnaemeka C. Iriemenam, Fehintola A. Ige, Stacie M. Greby, Ado G. Abubakar, Olumide Okunoye, Nwando Mba, Ndidi P. Agala, Alash'le Abimiku, McPaul I. Okoye, Laura C. Steinhardt, Rosemary Audu.

Resources: Nnaemeka C. Iriemenam, Stacie M. Greby, Augustine Mpamugo, Ado G. Abubakar, Ayuba B. Dawurung, Mudiaga K. Esiekpe, Nwando Mba, Ndidi P. Agala, Kristen A. Stafford, Alash'le Abimiku, Mahesh Swaminathan, McPaul I. Okoye.

Supervision: Nnaemeka C. Iriemenam, McPaul I. Okoye.

Validation: Nnaemeka C. Iriemenam, Fehintola A. Ige, Augustine Mpamugo, Ado G. Abubakar, Ayuba B. Dawurung, Mudiaga K. Esiekpe, Andrew N. Thomas, Mary U. Okoli, Samuel S. Awala, Blessing N. Ugboaja, Chicago C. Achugbu, Ifeanyichukwu Odoh, Felicia D. Nwatu, Temitope Olaleye, Loveth Akayi, Oluwaseun O. Akinmulero, Joseph Dattijo, Edewede Onokevbagbe, Mabel Uwandu, Maureen Aniedobe, Rosemary Audu.

Visualization: Augustine Mpamugo, Ado G. Abubakar, Ayuba B. Dawurung, Mudiaga K. Esiekpe, Andrew N. Thomas, Mary U. Okoli, Samuel S. Awala, Blessing N. Ugboaja, Chicago C. Achugbu, Ifeanyichukwu Odoh, Felicia D. Nwatu, Temitope Olaleye, Loveth Akayi, Edewede Onokevbagbe, Olumide Okunoye, Mabel Uwandu, Maureen Aniedobe, Rosemary Audu.

Writing – original draft: Nnaemeka C. Iriemenam.

Writing – review & editing: Nnaemeka C. Iriemenam, Fehintola A. Ige, Stacie M. Greby, Augustine Mpamugo, Ado G. Abubakar, Ayuba B. Dawurung, Mudiaga K. Esiekpe, Andrew N. Thomas, Mary U. Okoli, Samuel S. Awala, Blessing N. Ugboaja, Chicago C. Achugbu, Ifeanyichukwu Odoh, Felicia D. Nwatu, Temitope Olaleye, Loveth Akayi, Oluwaseun O. Akinmulero, Joseph Dattijo, Edewede Onokevbagbe, Olumide Okunoye, Nwando Mba, Ndidi P. Agala, Mabel Uwandu, Maureen Aniedobe, Kristen A. Stafford, Alash'le Abimiku, Yohhei Hamada, Mahesh Swaminathan, McPaul I. Okoye, Laura C. Steinhardt, Rosemary Audu.

References

1. WHO. Coronavirus disease 2019 (COVID-19) Situation Report– 94: https://www.who.int/docs/default-source/coronaviruse/situation-reports/20200423-sitrep-94-covid-19.pdf; 2020 [cited 2021 February 25]. Available from: https://www.who.int/docs/default-source/coronaviruse/situation-reports/20200423-sitrep-94-covid-19.pdf.
2. WHO. WHO Coronavirus Disease (COVID-19) Dashboard https://covid19.who.int/2022 [cited 2022 January 17]. Available from: https://covid19.who.int/.
3. WHO. Coronavirus disease (COVID-19) technical guidance: The Unity Studies: Early Investigation Protocols: https://www.who.int/emergencies/diseases/novel-coronavirus-2019/technical-guidance/early-investigations; 2020 [cited 2021 February 25]. Available from: https://www.who.int/emergencies/diseases/novel-coronavirus-2019/technical-guidance/early-investigations.
4. FDA. EUA Authorized Serology Test Performance: https://www.fda.gov/medical-devices/coronavirus-disease-2019-covid-19-emergency-use-authorizations-medical-devices/eua-authorized-serology-test-performance?utm_campaign=2020-05-07%20CDRH%20New&utm_medium=email&utm_source=Eloqua; 2021 [cited 2021 March 1]. Available from: https://www.fda.gov/medical-devices/coronavirus-disease-2019-covid-19-emergency-use-authorizations-medical-devices/eua-authorized-serology-test-performance?utm_campaign=2020-05-07%20CDRH%20New&utm_medium=email&utm_source=Eloqua.
5. WHO. Emergency use listing: https://www.who.int/teams/regulation-prequalification/eul; 2021 [cited 2021 March 9]. Available from: https://www.who.int/teams/regulation-prequalification/eul.
6. Yadouleton A, Sander AL, Moreira-Soto A, Tchibozo C, Hounkanrin G, Badou Y, et al. Limited Specificity of Serologic Tests for SARS-CoV-2 Antibody Detection, Benin. Emerg Infect Dis. 2021; 27(1). Epub 2020/12/03. https://doi.org/10.3201/eid2701.203281 PMID: 33261717; PubMed Central PMCID: PMC7774555.
7. Emmerich P, Murawski C, Ehmen C, von Possel R, Pekarek N, Oestereich L, et al. Limited specificity of commercially available SARS-CoV-2 IgG ELISAs in serum samples of African origin. Trop Med Int Health. 2021; 26(6):621–31. Epub 2021/03/06. https://doi.org/10.1111/tmi.13569 PMID: 33666297; PubMed Central PMCID: PMC8014856.
8. Schwarz NG, Mertens E, Winter D, Maiga-Ascofare O, Dekker D, Jansen S, et al. No serological evidence for Zika virus infection and low specificity for anti-Zika virus ELISA in malaria positive individuals among pregnant women from Madagascar in 2010. PLoS One. 2017; 12(5):e0176708. Epub 2017/05/17. https://doi.org/10.1371/journal.pone.0176708 PMID: 28510593; PubMed Central PMCID: PMC5433683.
9. Tan SS, Chew KL, Saw S, Jureen R, Sethi S. Cross-reactivity of SARS-CoV-2 with HIV chemiluminescent assay leading to false-positive results. J Clin Pathol. 2020. Epub 2020/09/11. https://doi.org/10.1136/jclinpath-2020-206942 PMID: 32907911.
10. Fotis C, Meimetis N, Tsolakos N, Politou M, Akinosoglou K, Pliaka V, et al. Accurate SARS-CoV-2 seroprevalence surveys require robust multi-antigen assays. Sci Rep 2021; 11:6614. https://doi.org/10.1038/s41598-021-86035-2 PMID: 33758278
11. Rosado J, Pelleau S, Cockram C, Merkling SH, Nekkab N, Demeret C, et al. Multiplex assays for the identification of serological signatures of SARS-CoV-2 infection: an antibody-based diagnostic and machine learning study. Lancet Microbe. 2021; 2(2):e60–e9. Epub 2021/02/02. https://doi.org/10.1016/S2666-5247(20)30197-X PMID: 33521709; PubMed Central PMCID: PMC7837364.
12. Nkuba Ndaye A, Hoxha A, Madinga J, Mariën J, Peeters M, Leendertz FH, et al. Challenges in interpreting SARS-CoV-2 serological results in African countries. Lancet Glob Health. 2021; 9(5):e588–e9. Epub 2021/02/21. https://doi.org/10.1016/S2214-109X(21)00060-7 PMID: 33609481; PubMed Central PMCID: PMC7906714.
13. WHO. COVID-19 Target product profiles for priority diagnostics to support response to the COVID-19 pandemic v.1.0: https://www.who.int/publications/m/item/covid-19-target-product-profiles-for-priority-

diagnostics-to-support-response-to-the-covid-19-pandemic-v.0.1; 2020 [cited 2021 February 25]. Available from: https://www.who.int/publications/m/item/covid-19-target-product-profiles-for-priority-diagnostics-to-support-response-to-the-covid-19-pandemic-v.0.1.

14. Ige F, Hamada Y, Steinhardt LC, Iriemenam NC, Uwandu M, Greby SM, et al. Validation of Commercial SARS-CoV-2 Immunoassays in a Nigerian Population. Microbiology Spectrum. 2021; 9:e00680–21. Epub Oct 6. https://doi.org/10.1128/Spectrum.00680-21 PubMed Central PMCID: PMC8510257. PMID: 34612691

15. Steinhardt LC, Ige F, Iriemenam NC, Greby SM, Hamada Y, Uwandu M, et al. Cross-reactivity of two SARS-CoV-2 serological assays in a malaria-endemic setting. J Clin Microbiol. 2021; 59(7):e0051421. Epub Jun 18. https://doi.org/10.1128/JCM.00514-21 PMID: 33853839

16. Luminex. xMAP® SARS-CoV-2 Multi-Antigen IgG Assay Package Insert: https://www.fda.gov/media/140256/download; 2020 [cited 2021 March 2]. Available from: https://www.fda.gov/media/140256/download.

17. Wilson EB. Probable inference, the law of succession, and statistical inference. Journal of the American Statistical Association. 1927; 22(158):209–12.

18. Manning JE, Duffy PE, Esposito D, Sadtler K. Material strategies and considerations for serologic testing of global infectious diseases. MRS Bull. 2021:1–5. Epub 20210913. https://doi.org/10.1557/s43577-021-00167-4 PMID: 34539056; PubMed Central PMCID: PMC8437333.

19. Klumpp-Thomas C, Kalish H, Drew M, Hunsberger S, Snead K, Fay MP, et al. Standardization of ELISA protocols for serosurveys of the SARS-CoV-2 pandemic using clinical and at-home blood sampling. Nature communications. 2021; 12(1):113. https://doi.org/10.1038/s41467-020-20383-x PMID: 33397956

20. Liu G, Rusling JF. COVID-19 Antibody Tests and Their Limitations. ACS Sens. 2021; 6(3):593–612. Epub 2021/02/06. https://doi.org/10.1021/acssensors.0c02621 PMID: 33544999; PubMed Central PMCID: PMC7885805.

21. Tso FY, Lidenge SJ, Pena PB, Clegg AA, Ngowi JR, Mwaiselage J, et al. High prevalence of pre-existing serological cross-reactivity against severe acute respiratory syndrome coronavirus-2 (SARS-CoV-2) in sub-Saharan Africa. Int J Infect Dis. 2021; 102:577–83. Epub 2020/11/12. https://doi.org/10.1016/j.ijid.2020.10.104 PMID: 33176202; PubMed Central PMCID: PMC7648883.

22. Dobano C, Vidal M, Santano R, Jimenez A, Chi J, Barrios D, et al. Highly Sensitive and Specific Multiplex Antibody Assays To Quantify Immunoglobulins M, A, and G against SARS-CoV-2 Antigens. J Clin Microbiol. 2021; 59(2). Epub 2020/11/01. https://doi.org/10.1128/JCM.01731-20 PMID: 33127841.

23. Marien J, Ceulemans A, Michiels J, Heyndrickx L, Kerkhof K, Foque N, et al. Evaluating SARS-CoV-2 spike and nucleocapsid proteins as targets for antibody detection in severe and mild COVID-19 cases using a Luminex bead-based assay. J Virol Methods. 2021; 288:114025. Epub 2020/11/24. https://doi.org/10.1016/j.jviromet.2020.114025 PMID: 33227340; PubMed Central PMCID: PMC7678438.

24. McBride R, van Zyl M, Fielding BC. The coronavirus nucleocapsid is a multifunctional protein. Viruses. 2014; 6(8):2991–3018. Epub 2014/08/12. https://doi.org/10.3390/v6082991 PMID: 25105276; PubMed Central PMCID: PMC4147684.

25. Anderson EM, Goodwin EC, Verma A, Arevalo CP, Bolton MJ, Weirick ME, et al. Seasonal human coronavirus antibodies are boosted upon SARS-CoV-2 infection but not associated with protection. Cell. 2021; 184(7):1858–64 e10. Epub 2021/02/26. https://doi.org/10.1016/j.cell.2021.02.010 PMID: 33631096; PubMed Central PMCID: PMC7871851.

26. Qiu M, Shi Y, Guo Z, Chen Z, He R, Chen R, et al. Antibody responses to individual proteins of SARS coronavirus and their neutralization activities. Microbes Infect. 2005; 7(5–6):882–9. Epub 2005/05/10. https://doi.org/10.1016/j.micinf.2005.02.006 PMID: 15878679; PubMed Central PMCID: PMC7110836.

27. Li K, Huang B, Wu M, Zhong A, Li L, Cai Y, et al. Dynamic changes in anti-SARS-CoV-2 antibodies during SARS-CoV-2 infection and recovery from COVID-19. Nat Commun. 2020; 11(1):6044. Epub 2020/11/29. https://doi.org/10.1038/s41467-020-19943-y PMID: 33247152; PubMed Central PMCID: PMC7699636.
